# Comparative Effects of Snoring Sound between Two Minimally Invasive Surgeries in the Treatment of Snoring: A Randomized Controlled Trial

**DOI:** 10.1371/journal.pone.0097186

**Published:** 2014-05-09

**Authors:** Li-Ang Lee, Jen-Fang Yu, Yu-Lun Lo, Ning-Hung Chen, Tuan-Jen Fang, Chung-Guei Huang, Wen-Nuan Cheng, Hsueh-Yu Li

**Affiliations:** 1 Department of Otorhinolaryngology-Head and Neck Surgery, Chang Gung Memorial Hospital at Linkou, School of Medicine, College of Medicine, Chang Gung University, Taoyuan, Taiwan, R.O.C.; 2 Sleep Center, Chang Gung Memorial Hospital at Linkou, Taoyuan, Taiwan, R.O.C.; 3 Department of Otolaryngology, Xiamen Chang Gung Hospital, Xiamen, Fujian Province, China; 4 Graduate Institute of Medical Mechatronics, Taiouan Interdisciplinary Otolaryngology Laboratory, Chang Gung University, Taoyuan, Taiwan, R.O.C.; 5 Department of Pulmonary and Critical Care Medicine, Chang Gung Memorial Hospital at Linkou, School of Medicine, College of Medicine, Chang Gung University, Taoyuan, Taiwan, R.O.C.; 6 Department of Laboratory Medicine, Chang Gung Memorial Hospital at Linkou, College of Medicine, Chang Gung University, Taoyuan, Taiwan, R.O.C.; 7 Graduate School of Recreation and Sports Management, Taipei Physical Education College, Taipei, Taiwan, R.O.C.; Hospital General Dr. Manuel Gea González, Mexico

## Abstract

**Background:**

Minimally invasive surgeries of the soft palate have emerged as a less-invasive treatment for habitual snoring. To date, there is only limited information available comparing the effects of snoring sound between different minimally invasive surgeries in the treatment of habitual snoring.

**Objective:**

To compare the efficacy of palatal implant and radiofrequency surgery, in the reduction of snoring through subjective evaluation of snoring and objective snoring sound analysis.

**Patients and Method:**

Thirty patients with habitual snoring due to palatal obstruction (apnea-hypopnea index ≤15, body max index ≤30) were prospectively enrolled and randomized to undergo a single session of palatal implant or temperature-controlled radiofrequency surgery of the soft palate under local anesthesia. Snoring was primarily evaluated by the patient with a 10 cm visual analogue scale (VAS) at baseline and at a 3-month follow-up visit and the change in VAS was the primary outcome. Moreover, life qualities, measured by snore outcomes survey, and full-night snoring sounds, analyzed by a sound analytic program (Snore Map), were also investigated at the same time.

**Results:**

Twenty-eight patients completed the study; 14 received palatal implant surgery and 14 underwent radiofrequency surgery. The VAS and snore outcomes survey scores were significantly improved in both groups. However, the good response (postoperative VAS ≤3 or postoperative VAS ≤5 plus snore outcomes survey score ≥60) rate of the palatal implant group was significantly higher than that of the radiofrequency group (79% *vs*. 29%, P = 0.021). The maximal loudness of low-frequency (40–300 Hz) snores was reduced significantly in the palatal implant group. In addition, the snoring index was significantly reduced in the radiofrequency group.

**Conclusions:**

Both palatal implants and a single-stage radiofrequency surgery improve subjective snoring outcomes, but palatal implants have a greater effect on most measures of subjective and objective snoring. Multi-stage radiofrequency surgery was not tested.

**Trial Registration:**

ClinicalTrials.gov NCT01955083

## Introduction

Recent publications have demonstrated reductions in snoring with several minimally invasive surgery (MIS) methods of the soft palate including radiofrequency (RF) surgery and palatal implant (PI) [1], [2]. Despite modest effects in the treatment of obstructive sleep apnea [1]–[4], patients often wish to receive MIS for habitual snoring. However, the efficacy in reducing snoring has mainly been determined by self-reported questionnaires in the past. Further, the definition of surgical success in snoring treatment has not been universally defined. To date, changes in snoring sound characteristics following MIS have not been demonstrated.

Many cohort studies and a few randomized controlled trials or clinical controlled trials have compared MIS with a placebo [5]–[9], different energy generators [10], different material rigidity [11], or different operative techniques [12], [13]. RF of the soft palate produces volumetric tissue reduction [14] and selective scar tissue [5] to reduce obstruction and induce stiffness. However, the RF energy delivered to the soft palate can be inadequate and may result in residual or recurrent snoring [7], [15]. PI can decrease palatal flutter by increasing the rigidity of the soft palate through implant identity and tissue fibrosis [16], [17]. In addition, PI can be chronically retained in the muscle layer of the soft palate thereby producing a long-term anti-snoring effect [18], [19]. Nevertheless, whether PI provides a better efficacy in the treatment of snoring than RF surgery is still unknown.

The primary aim of the current study was to compare the anti-snoring effect between PI and RF by subjective assessments in a randomized controlled parallel trial. The secondary aim was to explore and compare the acoustic changes in snoring sounds after PI and RF.

## Materials and Methods

The protocol for this trial and supporting CONSORT checklist are available as supporting information; see Protocol S1 and Checklist S1.

### Ethics Statement

This study was approved by the Institutional Review Board of Chang Gung Memorial Hospital and conducted according to the principles expressed in the Declaration of Helsinki. Signed informed consent was obtained from all patients. Full details of the trial protocol can be found in the Supplementary Appendix, available with the full text of this article at www.plosone.org.

### Participants and Setting

**Figure 1:**
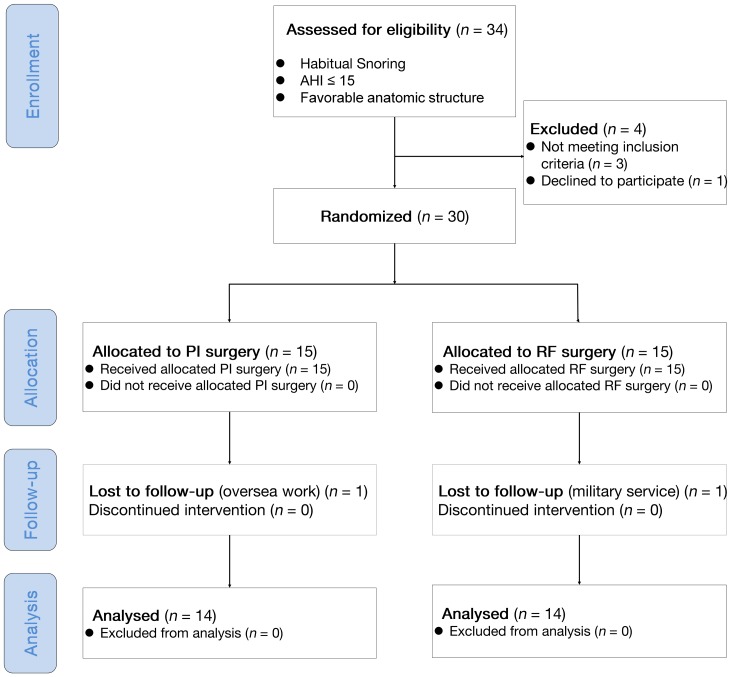
Schematic diagram (CONSORT 2010 flowchart diagram) summarizing the study design. AHI: apnea-hypopnea index. PI: palatal implant; RF, radiofrequency. SOS: snoring outcomes survey. VAS: visual analogue scale.

This study was a prospective, randomized, parallel-controlled, open labeled trial that was conducted from August 1, 2010 to July 30, 2012 in a tertiary medical center (Department of Otorhinolaryngology, Head and Neck Surgery, Chang Gung Memorial Hospital at Linkou, Taoyuan, Taiwan). The flowchart diagram following the CONSORT 2010 guideline demonstrated our study design (Figure 1). No important changes to methods (such as eligibility criteria) have been made after trial commencement. Thirty-four patients with habitual snoring and apnea-hypopnea index (AHI) ≤15 events/h confirmed by standard full-night polysomnography were prospectively recruited from October 1, 2010 to March 30, 2012 for the treatment of snoring by the two MIS methods. Standard level I polysomnography (Nicolet UltraSom System, Madison, WI, USA) was performed in the sleep laboratory to document sleep parameters in each patient. All respiratory events were scored as per standard criteria [20].

**Figure 2:**
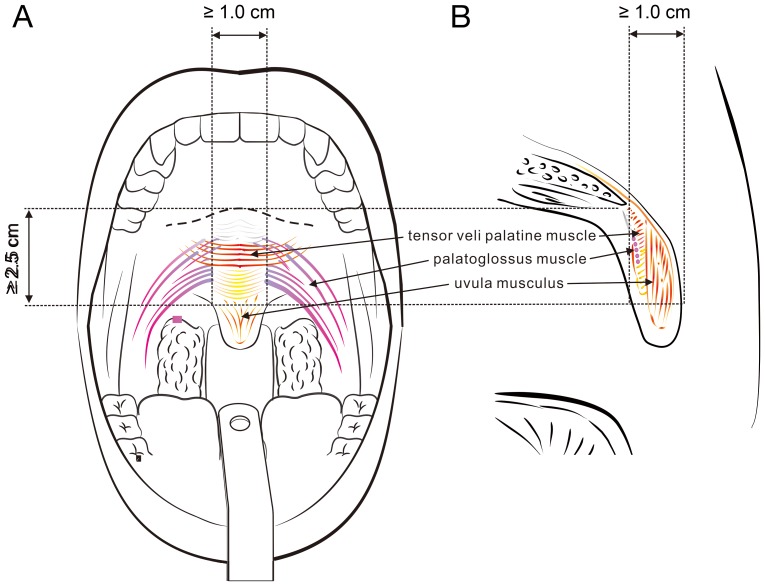
Favorable anatomy for the MIS of the soft palate in anti-snoring treatment. (A) Frontal view showing the longitudinal length from the uvular base to the hard palate-soft palate junction ≥ 2.5 cm and the lateral width of the uvular base ≥ 1.0 cm. (B) Lateral view illustrating the anterior-posterior width of the uvular base ≥ 1.0 cm.

The inclusion criteria were: a) age, 18–60 years; b) body mass index, ≤30 kg/m^2^; c) length of the soft palate (from the uvula base to the hard palate-soft palate junction) ≥2.5 cm and the width of the base of the uvular ≥1.0 cm (Figure 2). These criteria were basically in accordance with the criteria for a PI procedure as described in the literature [6], [9], [11], [16]. Patients were excluded if they had tonsillar hypertrophy (tonsil size ≥3), high tongue position (Friedman tongue position ≥4), retrognathia, craniofacial abnormalities, trismus, allergy to anesthetic or poorly controlled medical disorders such as hypertension, bleeding tendency, cardiovascular disorder, and stroke. Four patients were excluded due to ‘Not meeting inclusion criteria (*n* = 3)’ and ‘Declined to participate (*n* = 1)’.

Therefore, 30 participants were therefore enrolled in the present study. Subjective assessments of snoring and snoring sound recording were conducted after enrollment. The randomization procedure assured balanced design in subjective snoring severity and we stratified our subjects into two subgroups: ‘VAS >7’ (*n* = 12) and ‘VAS ≤7’ subgroups (*n* = 18). Computer-generated lists of random numbers were created using Random Number Generators of SPSS software for allocation of the participants and were stratified by center with a 1:1 allocation using a fixed block size of 6 (Rv. Uniform [0, 1]) in both subgroups. Participants were enrolled by four authors (LAL, YLL, NHC, and HYL). The allocation sequence was concealed from the researcher (CGH) before operation and the MIS procedure was performed by a single surgeon (HYL) who adhered to our computer-generated randomization protocol. Participants were randomly allocated to either PI or RF group (Figure 1). Half of the patients were randomized to the PI group and the remaining patients were randomized to the RF group. Subjective and objective assessments were re-measured 3 months following the MIS.

### Snoring Sound Assessment

#### Subjective snoring questionnaires

The snoring of all subjects was assessed by two subjective surveys: visual analogue scale (VAS) and snore outcomes survey (SOS) questionnaires. The participants, based on descriptions from their spouse or bed partner, were asked to estimate the severity of their snoring using a 10 cm VAS from 0 (no snoring) to 10 (very severe snoring, bed partner leaves the room). The SOS questionnaire was comprised of eight Likert-type items to comprehensively evaluate the duration, loudness, and frequency of snoring. The scale of the SOS was normalized from 0 (worst) to 100 (best) [21]. The Mandarin Chinese version of the SOS has been validated and repeatedly used as an outcome measure in obstructive sleep apnea patients [22].

#### Objective snoring sound analysis

We collected full-night snoring sounds of each subject in a standard sleep laboratory using a snore detection system as described previously [23]. An external measurement microphone (TEDS type 46AE, G.R.A.S. Corp., Holte, Denmark) was positioned 100 cm above the patient's head to record the snoring sounds in the sleep laboratory [23], [24]. Calibration of sound pressure level was performed before each test. The environmental sounds of the study room were recorded for 10 minutes as the background signal and analyzed. The recorded snoring sounds were detected by portable data cards (PXI 4462, National Instruments Corp., Austin, TX, USA) and processed by digital recording software (Sound & Vibration Toolkit for Labview, National Instruments Corp., Austin, TX, USA) at a sample rate of 44,100 Hz. The frequency power spectrum was created by fast Fourier transformation (range, 3.15 Hz–2,000 Hz).

After the patients had fallen asleep naturally and started to snore, we continually recorded the snoring sounds for 6 hours. Snoring sound signals were analyzed by the specially designed computer program Snore Map (Chang Gung Memorial Hospital, Taoyuan, Taiwan, R.O.C.). In accordance with an observer-blind study, the snoring sound recorder and the analyzer (JFY) were blind to the results of the randomization. The details of our snoring sound detection algorithm have been described elsewhere [23]. Using this method, we calculated the snoring index (SI [event/hour]), maximal sound intensity (Imax [dB]), mean sound intensity (Imean [dB]), peak sound frequency (Fpeak [Hz]), and mean sound frequency (Fmean [Hz]) in 4 different frequency domains (40 Hz–2,000 Hz [total-frequency, Total], 40 Hz–300 Hz [low-frequency, B1], 301 Hz–850 Hz [mid-frequency, B2], and 851 Hz–2,000 Hz [high-frequency, B3]).

In this study, we focused on the acoustic changes of Total- and B1-frequency domains due to the soft palate producing low frequency snores (<300 Hz) [25] and being operated on by the two different MIS.

### MIS

The patients underwent a session of either PI surgery or temperature-controlled RF surgery under local anesthesia on an outpatient basis. Before the MIS procedure, the palate was anesthetized by applying a topical 10% xylocaine spray followed by injection of a local anesthetic mixture of 2% lidocaine with diluted adrenaline (1:100,000). All of the study subjects were given antibiotics (ampicillin 500 mg q6h for 5 days) and analgesics (acetaminophen 500 mg q6h when required).

#### PI surgery

**Figure 3:**
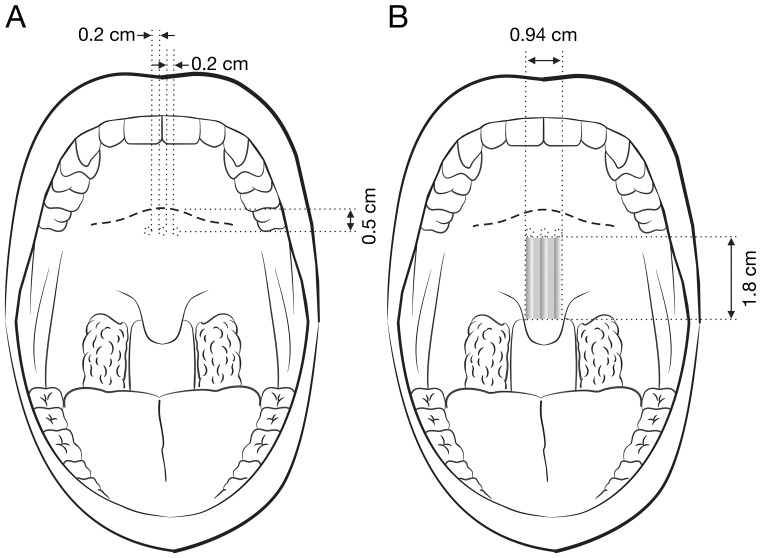
Palatal implant surgery. (A) Preoperative frontal view demonstrating the 3 operative sites of the soft palate (0.5 cm below the hard palate-soft palate junction; 0.2 cm between the midline and para-midline sites). (B) Postoperative frontal view showing the stiffened zone (grey zone) by the implant (1.8 cm×0.94 cm).

Using the delivery tool of the PI system (Pillar, Medtronic Inc., Jacksonville, FL, USA), the mucosa of the soft palate close to the hard palate-soft palate junction (approximate 0.5 cm) was punctured in the midline [26]. The needle was inserted to the uvular muscle and moved parallel to the curve of the soft palate towards the tip of the uvula. After reaching the insertion point, the implant was delivered steadily after which the needle was withdrawn. This process was repeated for the second and third implants in the bilateral para-midline with a 0.2 cm horizontal distance from the first implant (Figure 3).

#### RF surgery

**Figure 4:**
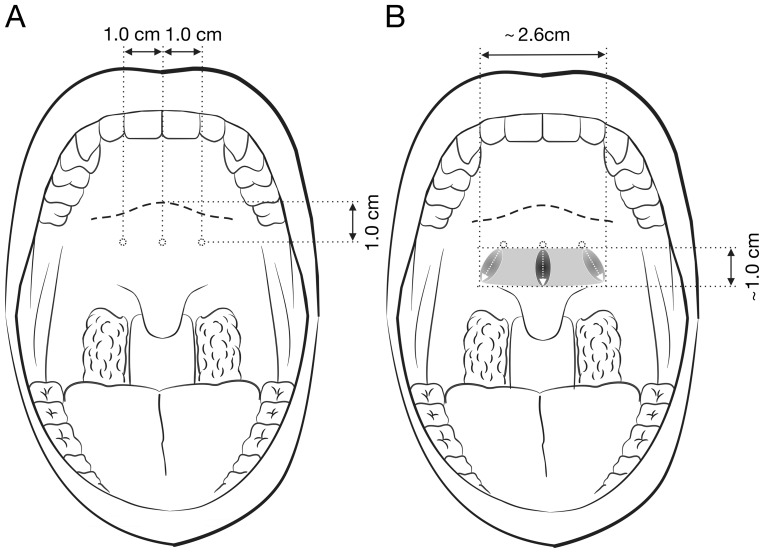
Radiofrequency surgery. (A) Preoperative frontal view illustrating the 3 operative sites of the soft palate (1.0 cm below the hard palate-soft palate junction; 1.0 cm between the midline and para-midline sites). (B) Postoperative frontal view demonstrating the stiffened zone (grey zone) by the RF energy (1.0 cm×2.6 cm).

RF energy was delivered via a generator (Somnus Model S2, Gyrus-ACMI Corporation, Maple Grove, MN, USA) with the power set to 10 watts and the maximal target temperature to 85°C. The needle electrode was inserted through the mucosa into the muscle layer at the entry points (approximately 1 cm below the hard palate-soft palate junction). The electrode was kept in place until 600 J had been delivered at the midline and 300 J at both para-midline sites (approximately 1 cm horizontal distance; Figure 4) [13], [27].

### Outcomes

The mean change in VAS before and after MIS was the primary outcome measurement. The mean changes in SOS and acoustic characteristics of snoring sound were the secondary outcome measurements. Postoperative ‘VAS ≤3’ was traditionally defined as ‘major response’ [28]. For a comprehensive profile of the outcomes, we further created another definition of ‘fine response’: ‘postoperative VAS ≤5 plus SOS ≥60’ post hoc in the present study. Accordingly, we compare a ‘good response’ rate, defined herein by a postoperative VAS ≤3 or postoperative VAS ≤5 plus SOS ≥60, between the PI and RF groups.

### Statistical Analysis

Statistical analyses were performed using SPSS software (version 17.0; SPSS, Inc., Chicago, IL, USA) and G*Power (version 3.1.5; University Kiel, Germany). For the relative small sample size of the present study, we analyzed all variables using non-parametric approaches. The sample size for this study was estimated using the primary outcome effects (VAS) in two previously published studies [6], [14]. Using a two-tailed Wilcoxon signed-rank test for calculating the sample size (normal parent distribution; effect size, 1.0; type I error, 0.05; power, 80%), we got a sample size of 11. For considering a 20% drop-out rate to fulfill the criteria of intention-to-treat analysis, we needed at least 14 participants to attend this study. Accordingly, we decided to enroll 30 patients with 15 in each group for showing the difference in mean before and after VAS scores. Two interim analyses were performed at the end of the first year during the trial: 1) the levels of significance maintained an overall *P* value of 0.05 and were calculated according to the O'Brien-Fleming stopping boundaries, and 2) this final analysis used a *Z* score of 1.985 with an associated *P* value of 0.0471 [29]. We planned to stop early if exceptional benefit or harm had been shown.

Further implementing the Kolmogorov-Smirnov test, distributions of patient clinical characteristics, subjective and objective snoring parameters were non-Gaussian and the descriptive statistics of these variables were presented as means (standard errors). Percentage (%) of change ([after value-before value]/[before value] × 100) in subjective snoring as well as in objective acoustic factors and differences between groups were analyzed with the Wilcoxon signed ranks test or the Mann-Whitney *U* test, as appropriate. Categorical variables were analyzed with the Fisher's exact test or the chi-square test. A *P* value of less than 0.05 was considered to be statistically significant.

## Results

### Subjects

Table 1 summarizes baseline anthropological data, polysomnographic parameters, and subjective snoring questionnaire scores for the entire study cohort. There were no significant differences in these parameters between the PI and RF groups. There were no protocol deviation in this study and all participants received the intended intervention after randomization. At the end of study (July 30, 2012), there were 28 patients completed the protocol; two study cases were not available for follow-up (one for oversea work and one for military service; Figure 1). Thus data from 28 patients (PI group, *n* = 14; RF group: *n* = 14) were available for the intention-to-treat analyses. Moreover, no significant complication was noted during the study period.

**Table 1:** Patient characteristics for the entire study cohort (*n* = 30).

	Overall	Palatal implant group	Radiofrequency group	Difference*	*P* value
	(*n* = 30)	(*n* = 15)	(*n* = 15)	(95% CI)	
**Gender** (% male [*n*])	90 (27)	100 (15)	80 (12)	20% (4% to 45%)	0.073
**Age** (years)	36.7 (1.7)	38.3 (3.1)	35.1 (1.9)	3.3 (−3.2 to 9.7)	0.288
**BMI** (kg/m^2^)	24.2 (0.5)	24.7 (0.8)	23.6 (0.7)	1.1 (−1.0 to 3.2)	0.983
**Neck circumference** (cm)	37.9 (0.6)	39.0 (0.9)	37.0 (0.9)	2.0 (−0.6 to 4.6)	0.391
**Tonsil size**	1.0 (0.03)	1.1 (0)	1.0 (0)	0.1 (−0.1 to 0.2)	0.317
**Friedman's tongue position**	2.4 (0.09)	2.3 (0.1)	2.4 (0.1)	−0.1 (−0.4 to 0.3)	0.710
**AHI** (events/hour)	7.8 (0.7)	7.9 (0.7)	7.7 (1.1)	0.2 (−2.6 to 3.1)	0.419
**PSG-SI** (events/hour)	290.2 (38.4)	307.1 (48.0)	273.3 (55.2)	33.8 (−126.0 to 193.6)	0.633
**VAS**	7.3 (0.4)	7.9 (0.5)	6.8 (0.6)	1.1 (−0.3 to 2.5)	0.384
**SOS**	40.5 (1.6)	40.4 (2.6)	40.5 (2.5)	−0.1 (−6.9 to 6.7)	0.934

Values were given as mean (standard error). AHI: apnea-hypopnea index. BMI: body mass index. CI: conference interval; PSG-SI: polysomnography-defined snoring index. SOS: snoring outcomes survey. VAS: visual analogue scale.

*Statistical analyses were implemented with the Mann-Whitney *U* test.

### Comparison of the Effects of PI and RF on Subjective Snoring Questionnaires

Changes in VAS scores after surgery were significant in both groups (PI group: 7.7 [0.4] *vs*. 4.4 [0.6], difference  = −3.3, 95% conference interval [CI], −5.0 to −1.6, *P* = 0.005; RF group: 6.6 [0.5] *vs*. 5.2 [0.4], difference = −1.4, 95% CI, −2.7 to −0.1, *P* = 0.022). Significant improvements in SOS scores were also noted in both groups (PI group: 39.4[2.2] *vs*. 62.2 [3.2], difference  =  22.8, 95% CI, 15.3 to 30.3, *P* = 0.002; RF group: 41.4 [2.4] *vs*. 54.4 [2.5], difference  =  13.0, 95% CI, 7.6 to 18.3, *P* = 0.001). Further, the mean change rate of VAS score in the PI group was significantly different from that in the RF group (−38.8% [9.0%] *vs*. −15.7% [7.2%], difference = 23.1%, 95% CI, −0.6% to 46.8%, *P* = 0.029). The mean change rate of SOS in the PI group was also significantly higher than that in the RF group (61.2% [10.1%] *vs*. 35.4% [7.7%], difference = 25.8%, 95% CI, −0.3% to 51.8%, *P* = 0.027).

Table 2 shows the results comparing the postoperative changes in subjective questionnaire scores after re-categorization between both groups. The rates of ΔVAS ≥1, ΔSOS ≥10, and response (ΔVAS ≥1 or ΔSOS ≥10) in both groups were not different. Twenty-nine percent of the PI group subjects reported mean postoperative VAS ≤3 (major response), and another 50% of the patients (fair response) complained of mild-to-moderate snoring (postoperative VAS ≤5) but had adequate functional outcomes (postoperative SOS ≥60). Only 7% of the RF group had major response after RF surgery, and 21% scored postoperative VAS ≤5 plus SOS ≥60 (fair response). Accordingly, the ‘good response’ rate was significantly higher in the PI group compared with the RF group (79% *vs.* 29%, difference  =  50%, 95% CI, 13% to 72%, *P* = 0.021).

**Table 2:** Comparison of the postoperative changes in subjective questionnaire scores between the palatal implant and radiofrequency surgery groups.

Subgroups, % (*n*)	Palatal implant group	Radiofrequency group	Ratio	Difference	*P* value
	(*n* = 14)	(*n* = 14)	(95% CI)	(95% CI)	
**ΔVAS ≥1**	93 (13)	64 (9)	1.44 (0.95–2.19)	29% (−3% to 55%)	0.165
**ΔSOS ≥10**	93 (13)	57 (8)	1.63 (1.01–2.62)	36% (3% to 61%)	0.077
**Postoperative VAS ≤3**	29 (4)	7 (1)	4.00 (0.51–31.46)	21% (−8% to 48%)	0.326
**Postoperative VAS ≤5 plus SOS ≥60**	71 (10)	29 (4)	2.50 (1.02–6.10)	43% (6% to 67%)	0.057
**Response (Either ΔVAS ≥1 or ΔSOS ≥10)**	93 (13)	71 (10)	1.30 (0.91–1.87)	21% (−8% to 48%)	0.326
**Good response (Ether postoperative VAS ≤3 or postoperative VAS ≤5 plus SOS ≥60)**	79 (11)	29 (4)	2.75 (1.15–6.58)	50% (13% to 72%)	0.021

CI: conference interval. SOS: snore outcomes survey. VAS: visual analogue scale.

### Comparison of the Effects of PI and RF on Objective Snoring Sound Analysis

Despite the randomized parallel-controlled trial nature of this study, some conditions were different between the two groups. The patients in the PI group had a significantly higher mean preoperative Total-Imax (70.9 [3.3] *vs*. 57.7 [2.1], difference = 13.2, 95% CI, 4.9 to 21.4, *P* = 0.004) and B1-Imax (63.8 [2.5] *vs*. 53.9 [2.0], difference = 9.8, 95% CI, 3.1 to 16.5, *P* = 0.014) than those in the RF group (Table 3).

**Table 3:** Objective snoring sound parameters at baseline for the entire study cohort (*n* = 30).

	Overall	Palatal implant group	Radiofrequency group	Difference*	*P* value
	(*n* = 30)	(*n* = 15)	(*n* = 15)	(95% CI)	
**Total domain (40 Hz–2000 Hz)**					
**SI** (events/hour)	116.3 (22.3)	116.3 (23.7)	116.4 (38.4)	−0.1 (−93.2 to 92.9)	0.135
**Imax** (dB)	64.3 (2.3)	70.9 (3.3)	57.7 (2.1)	13.2 (4.9 to 21.4)	0.004
**Imean** (dB)	49.1 (1.2)	50.8 (1.3)	47.4 (2.0)	3.4 (−1.4 to 8.3)	0.054
**Fpeak** (Hz)	580.3 (107.6)	543.3 (150.8)	617.3 (158.6)	−74.0 (−521.6 to 373.6)	0.709
**Fmean** (Hz)	101.9 (4.8)	101.4 (5.8)	102.3 (7.9)	−0.9 (−21.0 to 19.2)	0.868
**B1 domain (40 Hz–300 Hz)**					
**SI** (events/hour)	113.3 (22.3)	113.4 (23.6)	113.3 (38.5)	0.1 (−92.9 to 93.1)	0.135
**Imax** (dB)	58.9 (1.8)	63.8 (2.5)	53.9 (2.0)	9.8 (3.1 to 16.5)	0.004
**Imean** (dB)	46.0 (1.1)	47.2 (1.1)	44.8 (1.9)	2.4 (−2.1 to 7.0)	0.093
**Fpeak** (Hz)	219.0 (8.6)	217.3 (8.9)	220.7 (14.9)	−3.3 (−39.0 to 32.3)	0.934
**Fmean** (Hz)	100.0 (4.7)	98.0 (6.1)	101.9 (7.4)	−3.9 (−23.7 to 15.8)	0.494

Values were given as mean (standard error). AHI: apnea-hypopnea index. BMI: body mass index. CI: conference interval; PSG-SI: polysomnography-defined snoring index. SOS: snoring outcomes survey. VAS: visual analogue scale.

*Statistical analyses were implemented with the Mann-Whitney *U* test.

In the PI group, the change of mean B1-Imax (64.5 [1.9] *vs*. 56.7 [1.9], difference  =  −7.9, 95% CI, −16.4 to 0.7, *P* = 0.048) was significant, whereas changes of the other acoustic parameters were insignificant (Table 4). Although the mean B1-SI (121.2 [40.4] *vs*. 54.4 [31.4], difference  =  −66.9, 95% CI, −145.0 to 11.3, *P* = 0.041) was significantly reduced after surgery, the mean postoperative Total-Imax (58.3 [2.2] *vs*. 64.9 [3.3], difference = 6.7, 95% CI, 1.8 to 11.5, *P* = 0.016), Total-Imean (48.0 [2.0] *vs*. 56.3 [2.9], difference = 8.3, 95% CI, 2.3 to 14.2, *P* = 0.009), and B1-Imean (45.4 [2.0] *vs*. 51.6 [2.6], difference = 6.3, 95% CI, 1.1 to 11.4, *P* = 0.011) were significantly increased compared with the preoperative data in the RF group. As expected, the change rates of Total-Imax (−6.8% [5.9%] *vs*. 11.3% [3.8%], difference  =  −18.1%, 95% CI, −32.5% to −3.6%, *P* = 0.022), Total-Imean (−0.4% [5.6%]. *vs*. 18.2% [6.1%], difference  =  −17.9%, 95% CI, −34.9% to −0.9%], *P* = 0.027), B1-Imax (−9.4% [5.6%] *vs*. 5.6% [3.1%], [difference = −15.1%, 95% CI, −28.3%to −1.9%], *P* = 0.024), and B1-Imean (−4.6% [5.3%] *vs*. 14.7% [5.5%], difference = −19.3, 95% CI, −34.9% to −3.3%], *P* = 0.009) were significantly different between the PI group and RF group.

**Table 4:** Results of objective snoring sound parameters before and after treatment in the two groups.

	Palatal implant group (*n* = 14)			Radiofrequency group (*n* = 14)			
Parameters	Before	After	Mean percentage (%) of change*	Before	After	Mean percentage (%) of change*	Difference of mean percentage (%) of change** (95% CI)
**Total domain (40 Hz–2000 Hz)**							
**SI** (events/hour)	123.8 (24.8)	101.3 (28.8)	−5.9 (25.6)	124.6 (40.3)	73.9 (34.2)	−48.9 (22.1)	43.1 (−11.1 to 97.3)
**Imax** (dB)	72.1 (3.4)^A^	65.1 (1.8)	−6.8 (5.9)^C^	58.3 (2.2)^A^	64.9 (3.3)^B^	11.3 (3.8)^C^	−18.1 (−32.5 to −3.6)
**Imean** (dB)	50.9 (1.4)	50.6 (2.6)	−0.4 (5.6)^C^	48.0 (2.0)	56.3 (2.9)^B^	18.2 (6.1)^C^	−17.9 (−34.9 to −0.9)
**Fpeak** (Hz)	570.7 (161.9)	570.0 (146.6)	−6 (24.2)	651.5 (163.4)	477.9 (122.0)	−12.1 (26.2)	6.1 (−33.9 to 69.3)
**Fmean** (Hz)	100.7 (6.2)	99.6 (8.6)	−0.9 (8.7)	104.6 (8.1)	89.8 (12.7)	−10.9 (11.6)	10.0 (−18.0 to 41.5)
**B1 domain (40 Hz**–**300 Hz)**							
**SI** (events/hour)	120.8 (24.7)	116.2 (30.9)	−1.7 (29.5)	121.2 (40.4)	54.4 (31.4)^B^	−51.8 (22.5)	50.1 (−3.9 to 110.9)
**Imax** (dB)	64.5 (2.6)^A^	56.7 (1.9)^B^	−9.4 (5.6)^C^	54.4 (2.1)^A^	57.4 (2.6)	5.6 (3.1)^C^	−15.1 (−28.3 to −1.9)
**Imean** (dB)	47.2 (1.2)	44.7 (2.2)	−4.6 (5.3)^C^	45.4 (2.0)	51.6 (2.6)^B^	14.7 (5.5)^C^	−19.3 (−34.9 to −3.3)
**Fpeak** (Hz)	222.1 (9.6)	192.9 (15.9)	−10.9 (7.9)	226.4 (13.9)	142.1 (25.6)	−33.5 (11.4)	22.6 (−6.0 to 51.1)
**Fmean** (Hz)	97.0 (6.6)	95.2 (6.9)	−1.6 (8.4)	103.9 (7.6)	75.0 (9.4)	−24.8 (8.7)	22.2 (−1.5 to 51.3)

CI: conference interval. Fmean: mean sound frequency. Fpeak: peak sound frequency. Imax: maximal sound intensity. Imean: mean sound intensity.

## Discussion

This is the first study to compare the anti-snoring effects in two MIS of the soft palate, PI and RF, using both objective and subjective evaluations. Clinically, RF surgery is more widely used and investigated, while PI surgery has received increasing attention, although their efficacy in changing the objective characteristics of snoring sounds remains uncertain. Our results suggest that both PI and RF surgeries were safe and improved subjective snoring severity and functional outcomes; however there were differences in their effects on acoustic characteristics. Furthermore, PI surgery had a greater effect on a favorable surgical response than one-session of RF surgery in this randomized parallel-controlled trial.

Previous functional outcome studies of palatal MIS are limited, and most have used VAS score to measure the differences. The VAS for snoring assessment is a standardized, well-established tool to measure the subjective intensity of snoring. Many reports have shown that the patients who receive MIS for snoring usually have a high (>7) VAS score [1]–[4]. Nevertheless, only a few studies have demonstrated a MIS reduction in VAS snoring score to ≤3 [14], [26]–[28], [30]–[35]. Although MIS rarely cures snoring in subjective evaluation, it has been shown to effectively alleviate snoring in several randomized placebo-controlled trials [5], [6], [8], [9]. Further, VAS alone cannot reflect the whole profile of snoring and related consequences. Accordingly, full outcome analysis of MIS for snoring should include other snoring-related outcomes such as the Functional Outcomes of Sleep Questionnaire (FOSQ) [36] or SOS [21]. Steward et al. reported that PI surgery could improve FOSQ scores more significantly than placebo [8]. In our previous study, the SOS score significantly increased following one-session of RF surgery [14].

Theoretically, both PI and RF surgery stiffens the muscle layer of the soft palate in order to reduce the palatal flutter [16], [37]. Previous studies have indicated that patients with mild obstructive sleep apnea or simple snoring have peak intensities between 100 and 300 Hz (B1-domain) [23], [25], [38]. As expected, this study found that the two MIS mainly influenced B1-snoring sounds although in different dimensions. That is, the distinct mechanisms of the palatal rigidity can also alter the surrounding airway structures that can then produce different snoring sounds.

To the best of our knowledge, there are currently no reports on changes of the upper airway structure or changes in acoustic characteristics after PI surgery. Clinically, the shape and size of the uvula and soft palate do not change after PI procedure. However, polyethylene teralphate implants can be regarded as an extension of the hard palate to prop up and stiffen the soft palate in a rectangular area by approximately 1.8 cm (length) x 0.94 (0.18 + 0.2 + 0.18 + 0.2 + 0.18) cm (width; Figure 3B). It seems that the PI strengths the uvular muscle (Figure 1A) and consequently confines palatal flutter leading to the reduction in B1-Imax. Of note, VAS and SOS improved significantly in spite of insignificant reductions in other B1-domain parameters in the PI group.

In contrast, RF energy may affect the soft palate in a long oval area by approximately 1.0 cm (length) x 2.6 cm (width) based on our surgical technique (Figure 4B). The location of the RF treatment includes the musculus uvulae (midline position 600 J) and combination of levator veli palatini and palatopharyngeus (para-midline positions 300 J each). Bäck et al. used magnetic resonance imaging to evaluate soft palates that received two sessions of bipolar RF energy, and found that the scar tissue formation resulting in the T1-signal intensity of the soft palate increased significantly three months after treatment [26].

In this study, the subjective improvement rates in the VAS and SOS were significantly lower in the RF group than the PI group despite having the same inclusion criteria with regards to anatomy. Moreover, B1-SI decreased significantly without respect to the increase in B1-Imean. Since the length and width of the soft palate could not be significantly changed by RF surgery despite scaring of the soft palate [26], the non-operated part (at least 0.5 cm; 2.5 – 1.0 – 1.0 = 0.5) and insufficient longitudinal rigidity of the uvular muscle in one single session RF treatment cannot resist a higher inspiratory force resulting in louder B1-snores. In contrast, we presume that RF energy works on the tensor veli palatine and palatopharyngeus muscles in a horizontal direction (Figure 1A) and tenses up the soft palate to resist lower inspiratory force thereby decreasing the occurrence of B1-snores. These findings imply the necessity of more treatment sessions or multiple midline longitudinal injection sites in RF surgery of the soft palate to reduce the intensity of snoring, and the application of additional PI in the tensor veli palatine muscle to decrease SI.

The major limitation to this study is unbalanced randomization in Total-Imax despite insignificant differences in baseline anthropologic, symptomatic, and polysomnographic parameters between the two groups. This may be a methodological flaw in such a prospective randomized controlled trial, although this was not predicted before the study was undertaken. Moreover, the small sample size creates at least a few limitations: unable to do subgroup analyses, risk of unbalanced allocation of patients (i.e., imperfect randomization can be a consequence of the small sample size), and statistical difference between groups is seen only when there are large differences (i.e., smaller differences are not statistically significant, even if clinically important). A future study with a larger sample could overcome these limitations. Although our study indicated PI surgery may be more efficacious in the treatment of snoring for adult patients with simple snoring, it will be important to test the effects of this MIS technique with more anthropologically and ethnically diverse subjects in a larger sample.

## Conclusions

Both PI and RF alleviated subjective snoring intensity and improved snoring-related outcomes; however, the changes in magnitude and pattern in the acoustic parameters of snoring differed between these two MIS techniques. Our study indicated PI surgery may be more efficacious than single-stage RF surgery in the treatment of snoring for adult patients with simple snoring, it will be important to test the effects of this MIS technique with more anthropologically and ethnically diverse subjects in a larger sample. Further research evaluating the histoanatomical and acoustic changes within the soft palate to predict treatment outcomes is warranted.

## Acknowledgments

The authors would like to thank Mrs. Shin-Jao Lee, Department of Otorhinolaryngology-Head and Neck Surgery, Chang Gung Memorial Hospital at Linkou, Taoyuan, Taiwan, for assisting in data collection, and Mr. Yen-Sheng Chen and Mr. Ding-Li Wang, Graduate Institute of Medical Mechatronics, Taiouan Interdisciplinary Otolaryngology Laboratory, Chang Gung University, Taoyuan, Taiwan, for assisting in snoring sound acquisition.

## Supporting Information

Protocol S1 **Trial study protocol containing the complete and detailed plan (Chinese/English translation) for the conduct and analysis of the trial that the Institutional Review Board of Chang Gung Memorial Hospital approved before the trial began.**
(DOC)

Checklist S1 **CONSORT 2010 checklist compromising a 25-item checklist along with some brief descriptive text.**
(DOC)

## References

[1] Bäck LJ, Hytönen ML, Roine RP, Malmivaara AO (2009) Radiofrequency ablation treatment of soft palate for patients with snoring: a systematic review of effectiveness and adverse effects. Laryngoscope 119: 1241–1250. 19365852 10.1002/lary.20215

[2] Choi JH, Kim SN, Cho JH (2013) Efficacy of the Pillar implant in the treatment of snoring and mild-to-moderate obstructive sleep apnea: a meta-analysis. Laryngoscope 123: 269–276. 22865236 10.1002/lary.23470

[3] Stuck BA, Maurer JT, Hein G, Hörmann K, Verse T (2004) Radiofrequency surgery of the soft palate in the treatment of snoring: a review of the literature. Sleep 27: 551–555. 15164913 10.1093/sleep/27.3.551

[4] Franklin KA, Anttila H, Axelsson S, Gislason T, Maasilta P, et al (2009) Effects and side-effects of surgery for snoring and obstructive sleep apnea-a systematic review. Sleep 32: 27–36. 19189776 PMC2625321

[5] Stuck BA, Sauter A, Hörmann K, Verse T, Maurer JT (2005) Radiofrequency surgery of the soft palate in the treatment of snoring. A placebo-controlled trial. Sleep 28: 847–850. 16124664 10.1093/sleep/28.7.847

[6] Friedman M, Schalch P, Lin HC, Kakodkar KA, Joseph NJ, et al (2008) Palatal implants for the treatment of snoring and obstructive sleep apnea/hypopnea syndrome. Otolaryngol Head Neck Surg 138: 209–216. 18241718 10.1016/j.otohns.2007.10.026

[7] Bäck LJ, Liukko T, Rantanen I, Peltola JS, Partinen M, et al (2009) Radiofrequency surgery of the soft palate in the treatment of mild obstructive sleep apnea is not effective as a single-stage procedure: A randomized single-blinded placebo-controlled trial. Laryngoscope 119: 1621–1627. 19504550 10.1002/lary.20562

[8] Steward DL, Huntley TC, Woodson BT, Surdulescu V (2008) Palate implants for obstructive sleep apnea: multi-institution, randomized, placebo-controlled study. Otolaryngol Head Neck Surg 139: 506–510. 18922335 10.1016/j.otohns.2008.07.021

[9] Maurer JT, Sommer JU, Hein G, Hörmann K, Heiser C, et al (2012) Palatal implants in the treatment of obstructive sleep apnea: a randomised, placebo-controlled single-centre trial. Eur Arch Otorhinolaryngol 269: 1851–1856. 22228439 10.1007/s00405-011-1920-4

[10] Blumen MB, Chalumeau F, Gauthier A, Bobin S, Coste A, et al (2008) Comparative study of four radiofrequency generators for the treatment of snoring. Otolaryngol Head Neck Surg 138: 294–299. 18312874 10.1016/j.otohns.2007.11.008

[11] Skjøstad KW, Stene BK, Norgård S (2006) Consequences of increased rigidity in palatal implants for snoring: a randomized controlled study. Otolaryngol Head Neck Surg 134: 63–66. 16399182 10.1016/j.otohns.2005.10.006

[12] Bassiouny A, El Salamawy A, Abd El-Tawab M, Atef A (2007) Bipolar radiofrequency treatment for snoring with mild to moderate sleep apnea: a comparative study between the radiofrequency assisted uvulopalatoplasty technique and the channeling technique. Eur Arch Otorhinolaryngol 264: 659–667. 17294208 10.1007/s00405-007-0244-x

[13] Balsevičius T, Uloza V, Vaitkus S, Sakalauskas R, Miliauskas S (2013) Controlled trial of combined radiofrequency-assisted uvulopalatoplasty in the treatment of snoring and mild to moderate OSAS (pilot study). Sleep Breath 17: 695–703. 22743849 10.1007/s11325-012-0744-9

[14] Fang TJ, Li HY, Shue CW, Lee LA, Wang PC (2003) Efficacy of radiofrequency volumetric tissue reduction of the soft palate in the treatment of snoring. Int J Clin Pract 57: 769–772. 14686565

[15] Blumen MB, Vezina JP, Bequignon E, Chabolle F (2013) Snoring intensity after a first session of soft palate radiofrequency: predictive value of the final result. Laryngoscope 123: 1556–1559. 23625616 10.1002/lary.23800

[16] Ho WK, Wei WI, Chung KF (2004) Managing disturbing snoring with palatal implants: a pilot study. Arch Otolaryngol Head Neck Surg 130: 753–758. 15210558 10.1001/archotol.130.6.753

[17] Caples SM, Rowley JA, Prinsell JR, Pallanch JF, Elamin MB, et al (2010) Surgical modifications of the upper airway for obstructive sleep apnea in adults: a systematic review and meta-analysis. Sleep 33: 1396–1407. 21061863 10.1093/sleep/33.10.1396 PMC2941427

[18] Neruntarat C (2011) Long-term results of palatal implants for obstructive sleep apnea. Eur Arch Otorhinolaryngol 268: 1077–1080. 21298386 10.1007/s00405-011-1511-4

[19] Rotenberg BW, Luu K (2012) Four-year outcomes of palatal implants for primary snoring treatment: a prospective longitudinal study. Laryngoscope 122: 696–699. 22252926 10.1002/lary.22510

[20] Iber C, Ancoli-Israel S, Chesson AL, Quan SF (2007) The AASM manual for the scoring of sleep and associated events: Rules, terminology and technical specifications. 1st ed. Westchester: American Academy of Sleep Medicine.

[21] Gliklich RE, Wang PC (2002) Validation of the snore outcomes survey for patients with sleep-disordered breathing. Arch Otolaryngol Head Neck Surg 128: 819–824. 12117343 10.1001/archotol.128.7.819

[22] Chen NH, Li HY, Gliklich RE, Chu CC, Liang SC, et al (2002) Validation assessment of the Chinese version of the Snore Outcomes Survey. Qual Life Res 11: 601–607. 12206581 10.1023/a:1016337008763

[23] Lee LA, Yu JF, Lo YL, Chen YS, Wang DL, et al (2012) Energy types of snoring sounds in patients with obstructive sleep apnea syndrome: a preliminary observation. PLoS One 7: e53481. 23300931 10.1371/journal.pone.0053481 PMC3534069

[24] Ben-Israel N, Tarasiuk A, Zigel Y (2012) Obstructive apnea hypopnea index estimation by analysis of nocturnal snoring signals in adults. Sleep 35: 1299–1305. 22942509 10.5665/sleep.2092 PMC3413808

[25] Herzog M, Schmidt A, Bremert T, Herzog B, Hosemann W, et al (2008) Analysed snoring sounds correlate to obstructive sleep disordered breathing. Eur Arch Otorhinolaryngol 265: 105–113. 17680262 10.1007/s00405-007-0408-8

[26] Goessler UR, Hein G, Verse T, Stuck BA, Hormann K, et al (2007) Soft palate implants as a minimally invasive treatment for mild to moderate obstructive sleep apnea. Acta Otolaryngol 127: 527–531. 17453480 10.1080/00016480600951392

[27] Back LJ, Tervahartiala PO, Piilonen AK, Partinen MM, Ylikoski JS (2002) Bipolar radiofrequency thermal ablation of the soft palate in habitual snorers without significant desaturations assessed by magnetic resonance imaging. Am J Respir Crit Care Med 166: 865–871. 12231499 10.1164/rccm.2104110

[28] Powell NB, Riley RW, Troell RJ, Li K, Blumen MB, et al (1998) Radiofrequency volumetric tissue reduction of the palate in subjects with sleep-disordered breathing. Chest 113: 1163–1174. 9596289 10.1378/chest.113.5.1163

[29] Galgiani JN, Catanzaro A, Cloud GA, Johnson RH, Williams PL, et al (2000) Comparison of Oral Fluconazole and Itraconazole for Progressive, Nonmeningeal Coccidioidomycosis. A Randomized, Double-Blind Trial. Ann Intern Med 133: 676–686. 11074900 10.7326/0003-4819-133-9-200011070-00009

[30] Coleman SC, Smith TL (2000) Midline radiofrequency tissue reduction of the palate for bothersome snoring and sleep-disordered breathing: a clinical trial. Otolaryngol Head Neck Surg 122: 387–394. 10699816 10.1016/S0194-5998(00)70054-4

[31] Amorós-Sebastiá LI (2011) Radiofrequency treatment in simple snoring: tolerance, safety and results. Acta Otorrinolaringol Esp 62: 300–305. 21531359 10.1016/j.otorri.2011.03.002

[32] Blumen MB, Dahan S, Wagner I, De Dieuleveult T, Chabolle F (2002) Radiofrequency versus LAUP for the treatment of snoring. Otolaryngol Head Neck Surg 126: 67–73. 11821769 10.1067/mhn.2002.121017

[33] Iseri M, Balcioglu O (2005) Radiofrequency versus injection snoreplasty in simple snoring. Otolaryngol Head Neck Surg 133: 224–228. 16087019 10.1016/j.otohns.2005.04.018

[34] Sher AE, Flexon PB, Hillman D, Emery B, Swieca J, et al (2001) Temperature-controlled radiofrequency tissue volume reduction in the human soft palate. Otolaryngol Head Neck Surg 125: 312–318. 11593164 10.1067/mhn.2001.119141

[35] Terris DJ, Coker JF, Thomas AJ, Chavoya M (2002) Preliminary findings from a prospective, randomized trial of two palatal operations for sleep-disordered breathing. Otolaryngol Head Neck Surg 127: 315–323. 12402011 10.1067/mhn.2002.128345

[36] Nordgård S, Stene BK, Skjøstad KW (2006) Soft palate implants for the treatment of mild to moderate obstructive sleep apnea. Otolaryngol Head Neck Surg 134: 565–570. 16564373 10.1016/j.otohns.2005.11.034

[37] Weaver TE, Laizner AM, Evans LK, Maislin G, Chugh DK, et al (1997) An instrument to measure functional status outcomes for disorders of excessive sleepiness. Sleep 20: 835–843. 9415942

[38] Courey MS, Fomin D, Smith T, Huang S, Sanders D, et al (1999) Histologic and physiologic effects of electrocautery, CO2 laser, and radiofrequency injury in the porcine soft palate. Laryngoscope 109: 1316–1319. 10443841 10.1097/00005537-199908000-00025

