# Supplementary material for: Comparative Effects of Snoring Sound between Two Minimally Invasive Surgeries in the Treatment of Snoring: A Randomized Controlled Trial
Source: PLoS One. 2014 May 9;9(5):e97186. doi: 10.1371/journal.pone.0097186 (PMC4016275; doi:10.1371/journal.pone.0097186)
Supplement: Protocol S1 — Trial study protocol containing the complete and detailed plan (Chinese/English translation) for the conduct and analysis of the trial that the Institutional Review Board of Chang Gung Memorial Hospital approved before the trial began. (DOC) [file pone.0097186.s001.doc]

This study was approved by the Institutional Review Board of Chang Gung Memorial Hospital on April 09, 2010 (No.: 98-3870A3).

中文題目：睡眠呼吸障礙病患經微侵襲性手術後之鼾聲分析

中文關鍵字：阻塞性睡眠呼吸中止症，睡眠呼吸障礙，微侵襲性手術，鼾聲分析。

**背景：**打鼾的治療可分保守療法、微侵襲性手術與侵襲性手術。保守療法包括側睡、減重、改善鼻塞等，證據顯示，只有少部分病患能達到明顯改善。侵襲性手術以懸雍垂顎咽成形術為代表，此手術能顯著改善打鼾，但須全身麻醉且術後極度疼痛及其他合併症。微侵襲性手術以無線電波手術及軟顎植入術為代表，手術不須住院，局部麻醉下即可完成。報告顯示，微侵襲性手術能有效改善打鼾且幾乎沒有合併症。以往對於打鼾手術後效之評估：(1)多止於主觀的視覺量表分析(Visual Analog Scale)或問卷調查，極少對鼾聲作客觀分析以了解鼾聲之特性(如音量，頻率，次數，規律性等)。(2)未對主觀與客觀之結果作相關探討，以了解人體知覺感受與儀器檢查之差別。(3)更重要的是手術究竟改變了鼾聲的哪一個部份，是音量降低，或頻率提高，或次數減少，在過去並沒有報告作明確的說明。

**目的：**1、於睡眠實驗室建立有效的鼾聲收錄系統，對睡眠呼吸障礙病患，作睡眠檢查並收錄鼾聲，以了解鼾聲之音量、頻率、次數、規律性和模式，並與睡眠呼吸中止作相關探討。2、將客觀鼾聲分析之結果與病患和其睡眠拍檔對打鼾的主觀感受(問卷)做交叉比對，以了解主客觀評估之差異。3、比較微侵襲性手術治療前後，打鼾之主、客觀評估的變化，並比較兩種微侵襲性手術在降低鼾聲的成效，以深入了解不同的微侵襲性手術如何改善打鼾。

**方法：**第一年預定於門診中，收納30位經睡眠檢查證實為單純打鼾，或輕度睡眠呼吸中止症，且其解剖結構有利於微侵襲手術之睡眠呼吸障礙患者。病患符合條件，經醫師詳細告知研究流程及了解相關權利後，簽署告知同意書，正式成為本研究對象。病患首先於門診填寫打鼾問卷(Snore outcome survey)及視覺量表分析，並安排至本院睡眠中心，作整夜的鼾聲收錄。爾後病患接受隨機分配至無線電波手術或軟顎植入術。第二年，病患於術後三個月重複做問卷填寫、鼾聲收錄，以比較微侵襲性手術治療打鼾之客觀療效。

**預期成果：**本研究成果(1)能建立鼾聲測量系統，分析鼾聲頻譜， (2)了解主觀打鼾評估與客觀鼾聲分析之差異，(3)微侵襲性手術前後鼾聲之變化，(4)兩種不同手術方式對改善打鼾是否有所不同。

**Title:** Analysis of snore sound following minimal invasive surgery in sleep-disordered breathing patients.

**Key words:** Obstructive sleep apnea (OSA), sleep-disordered breathing, minimal invasive surgery, snore sound analysis

**Background**: The methods of anti-snore (treatment of snoring ) can be divided into conservative, invasive and minimal invasive treatment. Conservative treatment for snoring including sleep in one ride, body weight reduction and improvement of nasal obstruction may have limited results in some patients. Invasive treatment such as uvulopalatopharyngoplasty can significantly improve snoring with severe post-operative pain or potential major complications. Minimal invasive surgery involving radiofrequency and pillar implant demonstrates significantly improvement of snoring without major complication. Previous studies usually evaluated snoring through subjective measures such as visual analog scale visual analog scale. Rare reports analyzed snore sound instrumentally and no report demonstrates the correlation between subjective perception and objective assessment of snoring before and after surgical intervention.

**Purposes:** 1. Development of snore sound spectrum. 2. Exploration of the correlation between subjective perception and objective assessment of snoring. 3. Comparison of changes in snoring before and after minimal invasive surgery and between two kinds of MIS to have a understanding of surgical impact in subjective and objective measurement.

**Method:** We plan to enroll 30 subjects diagnosed by polysomnography as simple snoring or mild OSA with major complaint of snoring and favorable anatomic structure for minimal invasive surgery. All eligible subjects will be instructed the purpose, process and all related rights of this study and sign inform consent in outpatient clinic. Subjects start to complete Snore Outcome Survey (SOS, a validated questionnaire) and visual analog sure of snoring (VAS). Objective overnight snore sound recoding is arranged in sleep center. Subjects then receive minimal invasive surgery: radiofrequency or pillar implant of the soft palate by randomization (1:1). Both radiofrequency and pillar implant are common techniques in treating snoring and performed under local anesthesia as an outpatient procedure on sitting position. All subjects received repeated snore sound recording and completion of SOS and VAS three months after MIS.

**Outcomes:** (1) Development of snore sound spectrum in sleep-disorder breathing patients. (2) Correlation of parameters between snore sound recording (loudness, frequency, count, regularity, etc ) and clinical parameters. (3) Correlation between objective (snore sound analysis) and subjective (SOS,VAS) assessment of snoring. (4) Comparison of changes in snoring (particular in objective assessment) after radiofrequency or pillar implant. (5) Comparison of changes in snoring between radiofrequency and pillar implant.

**試驗計畫書 (Trial Protocol)**

(Note: English paragraphs of the trial protocol have been submitted to the Institutional Review Board of Chang Gung Memorial Hospital on February 2, 2010 and approved as’ Second Version of Trial Protocol’ on April 09, 2010; Chinese paragraphs of the ‘inform consent’ have been submitted to the Institutional Review Board of Chang Gung Memorial Hospital on February 2, 2010 and approved as’ Third Version of Inform Consent Form’ on April 09, 2010.)

**99/02/02 第二版 (February 2, 2010; 2^nd^ Version)**

**計劃主持人： 李學禹 Principle Investigator:** Hsueh-Yu Li,

**Title (試驗主題): Analysis of snore sound following minimal invasive surgery in sleep-disordered breathing patients. (睡眠呼吸障礙病患經微侵襲性手術後之鼾聲分析)**

**Background (簡介)**：

Snoring is an acoustic phenomenon produced by vibration of the palate and other structures in narrowed upper airway. The annoying sound affects the bed partners of up to 20 % of the population, and the incidence rises with advancing age. In addition to its relationship disharmony and social embarrassment, snoring is the cardinal symptom of obstructive sleep apnea (OSA). Heavy snoring, even without sleep apnea, has been implicated in the etiology of hypertension, ischemic heart disease, cerebrovascular accidents, and increased morbidity and mortality. (鼾聲的產生是因為上呼吸道狹窄，氣流經過這些狹窄區域流速會加快，使鬆弛的組織黏膜產生振動，因而出現鼾聲。打鼾的部位，以軟顎及懸壅垂最為常見，其他舌根、會厭、扁桃腺、後咽壁亦參與鼾聲的震動。打鼾的重要性可從兩方面分析：首先，打鼾容易對睡眠拍檔的產生困擾，嚴重時可能傷及婚姻與家庭，或使社交活動受到限制。其次，打鼾可能是罹患阻塞性睡眠呼吸中止症的重要指標。有打鼾症狀者，常倂有罹患高血壓或心血管疾病的機會亦較無打鼾者為高)

**Rationale:**

The methods of anti-snore (treatment of snoring) can be divided into conservative, invasive and minimal invasive treatment. Conservative treatment for snoring including sleep in one ride, body weight reduction and improvement of nasal obstruction may have limited results in some patients. Invasive treatment such as uvulopalatopharyngoplasty can significantly improve snoring with severe post-operative pain and potential major complications. Minimal invasive surgery (MIS) involving radiofrequency [Powell, 1998; Stuck, 2003; Fang 2003; Stuck, 2005] and pillar implant [Friedman, 2006; Friedman, 2006; Skjostad, 2006; Goessler, 2007; Friedman, 2008] has demonstrated significant improvement of snoring without major complication.

Previous studies usually evaluated snoring through subjective measures such as visual analogue scale (VAS) [Powell, 1998; Stuck, 2003; Fang 2003; Stuck, 2005; Friedman, 2006; Friedman, 2006; Skjostad, 2006; Goessler, 2007; Friedman, 2008]. Rare reports analyzed snore sound instrumentally and no report demonstrates the correlation between subjective perception and objective assessment of snoring before and after surgical intervention.

**Purposes of this study (試驗目的):**

1. Analyses of the characteristics of snore sound and its association to sleep apnea. (於睡眠實驗室建立有效的鼾聲收錄系統，對睡眠呼吸障礙病患，作睡眠檢查並收錄鼾聲，以了解鼾聲之音量、頻率、次數、規律性和模式，並與睡眠呼吸中止作相關探討。)
2. Exploration of the correlation between subjective perception and objective assessment of snoring. (將客觀鼾聲分析之結果與病患和其睡眠拍檔對打鼾的主觀感受(問卷)做交叉比對，以了解主客觀評估之差異。)
3. Comparison of changes in snoring before and after minimal invasive surgery and between two kinds of MIS to have a understanding of surgical impact in subjective and objective measurement. (比較微侵襲性手術治療前後，打鼾之主、客觀評估的變化，並比較兩種微侵襲性手術在降低鼾聲的成效，以深入了解不同的微侵襲性手術如何改善打鼾。)

**Method (試驗方法與程序說明):**

The sample size for this study was estimated using the outcome effects in two previously published studies [Friedman 2008; Fang 2003]. Using a two-tailed test, a type I error of 0.05, an effect size of 80% and a 20% drop-out rate to fulfill the criteria of intention-to-treat analysis, 30 patients with 15 in each group were calculated to show the difference with a power of 0.8 (G*Power , version 3.1.5; University Kiel, Germany).

In the first year, we plan to enroll 30 subjects diagnosed by polysomnography as simple snoring or mild OSA with major complaint of snoring and favorable anatomic structure for MIS. Subjects start to complete Snore Outcome Survey (SOS, a validated questionnaire) and VAS of snoring. (第一年，預定於門診中，收納30位主訴打鼾且非中、重度睡眠呼吸中止症，並有上呼吸氣道阻塞型態適合微侵襲性手術之睡眠呼吸障礙患者，同時患者需有意願接受手術治療。病患於門診當日需完成病史詢問、理學檢查、與鼾聲問卷調查(打鼾問卷及視覺量表)。

The inclusion criteria were: a) age, 18-60 years; b) body mass index, ≤30 kg/m^2^; c) length of the soft palate (from the uvula base to the hard palate-soft palate junction) ≥2.5 cm and the width of the base of the uvular ≥1.0 cm (Figure 2). These criteria were basically in accordance with the criteria for a PI procedure as described in the literature (納入標準：（1）年齡為20至60歲之患者；（2）上呼吸道解剖結構狹窄部位為口咽腔，例如：軟腭或懸雍垂過度肥厚或鬆弛（3）睡眠檢查證實為單純打鼾或輕度睡眠呼吸中止症。(4)有參與意願。).

Patients were excluded if they had tonsillar hypertrophy (tonsil size ≥3), high tongue position (Friedman tongue position ≥3), retrognathia, craniofacial abnormalities, trismus, allergy to anesthetic or poorly controlled medical disorders such as hypertension, bleeding tendency, cardiovascular disorder, and stroke. (排除標準：（1）上呼吸道解剖結構不利顎咽手術，例如：舌根部位過高、下巴內縮、顱顏骨架異常、張口困難、黏膜下纖維化、或曾接受過頭頸部放射線治療；（2）曾經接受口腔手術，例如懸壅垂顎咽成型術、雷射軟腭懸雍垂成型術、或口咽腫瘤切除手術；和（3）身體一般狀況不適合局部麻醉或手術者，例如：高血壓，出血性疾病、心血管疾病、中風、或病態性肥胖。)

All eligible subjects will be instructed the purpose, process and all related rights of this study and sign inform consent in out patient department. (病患符合條件，經醫師詳細告知研究目的、流程、手術方法及了解相關權利後，簽署告知同意書，正式成為本研究對象。)

Objective overnight snore sound recoding is performed in sleep center. Subjects then receive MIS: radiofrequency or pillar implant of the soft palate by randomization (1:1 allocation). A computer-generated list of random numbers was created using Random Number Generators (SPSS software, version 17.0; SPSS, Inc., Chicago, IL, USA) for allocation of the participants and was stratified by center with a 1:1 allocation (Rv. Uniform [0, 1]). (病患首先安排於桃園分院睡眠中心，在自然睡眠下作整夜的鼾聲收錄。爾後病患接受隨機分配(分配比例為1:1)進行微侵襲性軟顎手術(無線電波術或軟顎植入術)。)

We used three external measurement microphones (TEDS type 46AE, G.R.A.S. Corp., Holte, Denmark), positioned 100 cm above the patient’s head, to record snoring sounds at the sleep laboratory. The microphone detected sound frequencies between 3.15 and 20,000 Hz. A sound pressure level calibration was performed using a sound calibrator (B&K4321, Brüel & Kjær Corp., Nærum, Denmark) before each test. The intensities of the recorded snoring sounds were collected by portable data cards (PXI 4462, National Instruments Corp., Austin, TX, USA) and processed by digital recording software (Sound & Vibration Toolkit for Labview, National Instruments Corp., Austin, TX, USA) at sample rate of 44,100 Hz. The frequency power spectrum was created by fast Fourier transformation (range, 3.15 Hz-2,000 Hz). A snore detection system was designed to separate the snore episodes from environmental (machines, door opening, moving furniture, etc.) and other biological noises (body movements, oral communications, coughs, etc.). First, recording the environmental sounds of 10 minutes in a study room (silent sleep laboratory) were sampled and analyzed at the beginning of each test, and we found the highest intensity of background noises were relatively constant and occurred between 3.15 Hz and 40 Hz. Using a high-pass filter technique, the interference of background noises and snore episodes was reduced. Second, a snoring sound detection algorithm was designed based on an adaptive energy threshold. These “meaningful snoring events” were further analyzed in the present study. (本研究將使用3 支監測麥克風測量，麥克風(TEDS type 46AE, G.R.A.S. Corp., Holte, Denmark)架設在受試者口部正上方1 公尺。另外兩支麥克風將分別架設在平行受試者耳朵左右方1 公尺處，以確保能完全收錄受試者在不同睡姿時所發出的鼾聲。將利用聲音擷取軟體(Sound & Vibration Toolkit for Labview, National Instruments Corp., Austin, TX, USA)配合測量級麥克風（頻率範圍：3.15-20,000 Hz），在實驗進行前會以聲壓校正器(B&K4321, Brüel & Kjær Corp., Nærum, Denmark)來進行麥克風的校正，確認所收集到的聲音為正確無誤：聲壓校正器所釋放音頻為1000 Hz 且其聲壓為94 dBSPL，再將個別之麥克風與聲壓校正器進行比對，若量測之頻率為1000 Hz 且聲壓值為94 dBSPL，則完成麥克風之校正。最後利用聲音訊號本身的物理特性，經由各種轉換，加以量化分析，以找出鼾聲訊號之頻譜。再利用背景噪音（十分鐘）與鼾聲之間的差異，來判斷自然睡眠中的鼾聲。整夜鼾聲錄製完畢之後，利用聲音處理軟體及FFT(Fast Fourier Transform)原理進行轉換，畫面呈現之鼾聲頻譜將以縱軸為聲壓（sound pressure）及橫軸為頻率（frequency）來表示。聲音檔則會以wma 來做儲存。所測得的鼾聲錄音檔，則以點資料輸出，再將點資料匯入自行以撰寫的鼾聲分析程式，並將背景噪音點資料匯入，且以鼾聲聲壓高於背景噪音10 dBSPL 之SNR(signal-to-noise-ratio)來區分其為背景噪音或鼾聲。)

Both radiofrequency and pillar implant are common techniques in treating snoring and are performed on sitting position under local anesthesia as an out patient procedure. Radiofrequency energy (Somnus® Model S2, Gyrus-ACMI Corporation, Maple Grove, MN, USA) was delivered after inserting the needle electrode through the mucosa into the muscle layer at the entry points. The electrode was kept in place until 600 J had been delivered at the midline and 300 J at both para-midline sites using temperature control (≤ 85°C). This method can stiff the soft palate, decrease its vibration, reduce its volume, and increase the retropalatal space. Pillar implant procedure can be performed as radiofrequency surgery without delivering energy. Using the delivery tool of the pillar implant system (Pillar®, Medtronic Inc., Jacksonville, FL, USA), the mucosa of the soft palate close to the hard palate-soft palate junction was punctured in the midline. The needle was inserted to the uvular muscle and moved parallel to the curve of the soft palate towards the tip of the uvula. After reaching the insertion point, the PET implant was delivered steadily after which the needle was withdrawn. This process was repeated for the second and third implants in the bilateral para-midline. After approximate 15-minute operation, all of the study subjects will be measured blood pressure, take rest for minutes, and go home if no obvious discomfort). (這兩種手術都是門診手術，病患於坐姿下，先接受局部麻醉，再將微侵襲性手術的手機(hand piece)前端之針頭插入軟顎肌肉層。無線電波手術在此時作溫度控制下釋出能量，將組織加熱，達到軟顎變硬、振動變小，體積縮小，顎後空間變大的目的。軟顎移入術在手術方式與無線電波相似，差別在不釋出能量，而在手機針頭抽出時，同時把聚乙烯纖維推入軟顎預定位置來支撐軟顎(視同硬顎的延伸)，達到避免塌陷，減少振動，進而減輕打鼾之目的。兩種微侵襲性手術時間都大約15分鐘，術後病患量血壓，略作休息後若無不適即可離開醫院。)

Both the MIS techniques improve snoring by submucosal scarring and fibrosis. The clinical difference of these techniques is the radiofrequency surgery can be extensively applied in other anatomical locations such as inferior turbinate, tongue base, and tonsil. (兩種微侵襲性手術皆經由黏膜下結疤與纖維化來達到改善打鼾之目的。兩者之臨床差異在於無線電波手術還可以應用於下鼻甲、舌根與扁桃體，作用部位較廣泛。)

The alternative treatment of snoring is ‘laser-assisted uvulopalatoplasty’ that applies laser to cauterize the soft palate to produce scar tissue for reducing soft palate vibration and snoring. However, this surgery may result in extremely severe postoperative pain, narrowing retropalatal space, and worsening sleep apnea; therefore, laser-assisted uvulopalatoplasty is seldom applied to treat snoring in the present time. (類似上述兩種手術方式，在過去曾被廣泛使用的是雷射手術。藉由雷射直接燒灼軟顎造成疤痕組織來減少軟顎振動，達到減輕打鼾的效果。但因術後極度疼痛，且可能導致顎後空間變小，使睡眠呼吸中止惡化，現已少被使用。)

All subjects receive repeated snore sound recording and completion of SOS and VAS three months after MIS for comparison. (病患將於無線電波術或軟顎植入術後三個月重複接受自然睡眠下整夜鼾聲收錄及打鼾問卷和視覺量表填寫。)

**Contingencies for adverse outcomes (可能產生之副作用及危險):**

Problems that you may suffer are (您將可能遭遇的問題):

1. Mild swelling of the soft palate and discomfort during swallowing after surgery. These pharyngeal discomforts can happen in the first day and resolve within several days, and may make no significant influence on your diary life activity. (微侵襲性手術後第一天，可能有輕微的軟顎腫脹及吞嚥不適，這些咽喉症狀在幾天內會消退，不會影響您的日常生活。)
2. Occasionally, some patients may have dislodged of the implanted material from the soft palate. The dislodged material can be removed in the clinic and the patient may be receive another pillar implant procedure. (少數接受軟顎植入術之患者，植入物可能部份會脫出於軟顎，此時醫師會在門診中將其取出，並安排再做補充植入。)
3. Although the snoring sound detection system is design to measure the snoring without contacting the subjects and has no known side effect/risk to the participants, however, some patients may have insomnia possibly due to different sleeping environment. (鼾聲測量系統為非接觸式設計，並不會對受試者產生副作用或危險，惟以往部分病患可能因不同於居家之床位而有失眠現象。)

**Outcomes:**

The following flow chart may elucidate different outcomes derived from individual parts of protocol.


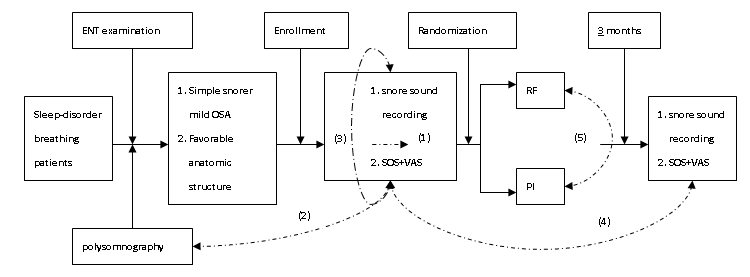


1. Development of snore sound spectrum in sleep-disorder breathing patients.
2. Correlation of parameters between snore sound recording (loudness, frequency, count, regularity, etc ) and polysomnography (apnea / hypopnea index, etc ). (Spearman correlation)
3. Correlation between objective (snore sound analysis) and subjective (SOS,VAS) assessment of snoring. (Spearman correlation)
4. Comparison of changes in snoring (particular in objective assessment) after radiofrequency or pillar implant. (Wilcoxon signed ranks test)
5. Comparison of changes in snoring between radiofrequency and pillar implant. (Wilcoxon signed ranks test)

**Clinical significance (預期試驗效果):**

The anticipated outcomes include（本研究預期的成果能包括）：

1. The developed model of snore sound analysis can be used to screen snorer for standard polysomonograpy in highly suspected patient of OSA to reduce long waiting list and medical expenditure. （建立鼾聲測量系統以分析鼾聲頻譜。）
2. The link between objective characteristics and subjective perception of snoring may result in a further understanding of the disturbance from snore sound to human. （有效評估微侵襲性手術對改善打鼾之主、客觀成果。）
3. Demonstration in the objective change of snore sound after MIS on the soft palate. Further elucidation of the potential difference in snore reduction from two difference surgical technique and mechanism (scarring effect in radiofrequency vs. tenting effect in pillar implant). （比較兩種微侵襲性手術對改善打鼾是否有所不同。)

**Other possible treatment modalities (其他可能之治療方式及說明):**

The other possible treatment modalities for the study subjects include life style modification (such as early sleep, daytime exercise, appropriate resting, and free from alcohol drinking before sleep), body weight reduction, oral appliance, and nasal continuous positive airway pressure. However, this study aim to perform MIS for anti-snore treatment, the participants should attend our study after fitting the inclusion and exclusion criteria. (本研究之受試者其他可能接受的睡眠呼吸障礙治療包括：生活作息的調整（例如早睡、多日間運動、勿過度勞累、勿睡前飲酒等等）、睡眠姿勢的改變（例如側睡）、減重、配戴口腔矯正牙套或、配戴連續型陽壓呼吸器。但本研究的著重以微侵襲性手術來進行鼾聲外科手術治療，受試者需符合納入與排除標準方得參加。)

**Potential problems:**

The major problem in this study is to enroll enough valid subjects in limited period because：

1. Most snoring patients visiting (or referred to) medical center are moderate or severe OSA patients with relatively unfavorable anatomy for MIS.
2. Patients already received overnight polysomnography may be reluctant to accept an overnight snore sound recording examination.
3. Patients are likely to decline follow-up overnight examination for snore sound recording especially in significantly improved cases.

**References:**

Fang TJ, Li HY*, Lee, LA, Wang PC. Efficacy of radiofrequency volumetric tissue reduction of the soft palate in the treatment of snoring. Int J Clin Prac 2003; 57: 769-772.

Friedman M, Vidyasagar R, Bliznikas D, Joseph NJ. Patient selection and efficacy of Pillar implant technique for the treatment of snoring and obstructive sleep apnea/hypopnea syndrome. Otolaryngol Head Neck Surg 2006;134:187-96.

Friedman M, Schalch P, Joseph NJ. Palatal stiffening after failed uvulopalatopharyngoplasty with the Pillar Implant System. Laryngoscope 2006;116:1956-61.

Friedman M, Schalch P, Lin HC, Kakodkar KA, Joseph NJ, et al. (2008) Palatal implants for the treatment of snoring and obstructive sleep apnea/hypopnea syndrome. Otolaryngol Head Neck Surg 138:209-216.

Goessler UR, Hein G, Verse T, et al. Soft palate implants as a minimally invasive treatment for mild to moderate obstructive sleep apnea. Acta Otolaryngol 2007;127:527-31.

Powell NB, Riley RW, Troell RJ, et al. Radiofrequency volumetric tissue reduction of the palate in subjects with sleep-disordered breathing. Chest 1998;113:1163-74.

Skjostad KW, Stene BK, Norgard S. Consequences of increased rigidity in palatal implants for snoring: a randomized controlled study. Otolaryngol Head Neck Surg 2006;134:63-6.

Stuck BA, Starzak K, Verse T, et al. Complications of temperature controlled radiofrequency volumetric tissue reduction for sleep-disordered breathing. Acta Otolaryngol 2003;123:532-5.

Stuck BA, Sauter A, Hormann K, et al. Radiofrequency surgery of the soft palate in the treatment of snoring. A placebo-controlled trial. Sleep 2005;28:847-50.
